# Early diving behaviour in juvenile penguins: improvement or selection processes

**DOI:** 10.1098/rsbl.2016.0490

**Published:** 2016-08

**Authors:** Florian Orgeret, Henri Weimerskirch, Charles-André Bost

**Affiliations:** Centre d'Etudes Biologiques de Chize UMR 7372 du CNRS-Universite de La Rochelle, 79360 Villiers-en-Bois, France

**Keywords:** juveniles, ontogeny, diving behaviour, penguins, bio-logging, tracking

## Abstract

The early life stage of long-lived species is critical to the viability of population, but is poorly understood. Longitudinal studies are needed to test whether juveniles are less efficient foragers than adults as has been hypothesized. We measured changes in the diving behaviour of 17 one-year-old king penguins *Aptenodytes patagonicus* at Crozet Islands (subantartic archipelago) during their first months at sea, using miniaturized tags that transmitted diving activity in real time. We also equipped five non-breeder adults with the same tags for comparison. The data on foraging performance revealed two groups of juveniles. The first group made shallower and shorter dives that may be indicative of early mortality while the second group progressively increased their diving depths and durations, and survived the first months at sea. This surviving group of juveniles required the same recovery durations as adults, but typically performed shallower and shorter dives. There is thereby a relationship between improved diving behaviour and survival in young penguins. This long period of improving diving performance in the juvenile life stage is potentially a critical period for the survival of deep avian divers and may have implications for their ability to adapt to environmental change.

## 1. Introduction

Foraging efficiency is thought to be a major cause of juvenile mortality [1–3] and is likely influenced by learning and foraging experience [3,4]. Early life mortality may be also explained by physiological capacities, whereby poor quality individuals die early and higher quality individuals survive to recruitment [2,3]. Unfortunately, early life stages of long-lived species are poorly understood despite their importance for recruitment to the reproductive life stage and thus the maintenance of healthy population levels [5]. Understanding the factors that affect the development of foraging skills is thus particularly important, especially in longed-lived marine species where little research has been done due to the logistical challenges of tracking juveniles [6].

Seabird juveniles are particularly difficult to monitor due to their large-scale dispersion over months. This is especially true for fledged penguins that disperse at sea for several months or years before returning to their natal colony [7,8]. Penguins are the most specialized of all seabirds for marine life and are one of the most abundant marine top predators in the Southern Hemisphere. They are considered sentinels of ocean health [9] and are deep divers [10]. Their propensity to capture prey during deep dives combined with an extended period of development provides an opportunity to determine the relationship between diving performance, physiological capacity and survival. However, few studies have addressed the dispersion of juvenile penguins (e.g. [7,11]) and only two studies with small sample size and short durations have analysed diving data [11,12].

In this study, we examined the diving performances of juvenile and non-breeder adult king penguins during the breeding season which were not constrained by the need to come back to the colony for breeding duties. We tested the hypothesis that improvement in foraging performances during early life was related to survivorship. We expected juveniles to be less efficient foragers than adults and would incrementally progress towards reaching adult levels of foraging capability (improvement in accordance with the ‘constraint hypothesis’ [2]). We also predicted an inter-individual variability in the progression of diving abilities (‘selection hypothesis’ [2]), with some individuals quickly progressing to adult skill levels, while others would underperform and die early. Thus, the foraging efficiency of some individuals (as measured by diving depths, durations and recovery times) was expected to decrease with time, such that individuals would not be able to reach the profitable prey depths [10].

## 2. Material and methods

Seventeen randomly selected king penguin juveniles and five non-breeder adults from the ‘Baie du Marin’ colony (Possession Island, Crozet Islands, South Indian Ocean) were equipped with SPLASH tags (Wildlife Computers, Redmond, WA, USA) in two different years. Table 1 summarizes the data on equipment date and monitoring duration of diving behaviour. The birds were equipped following standardized methods described in the electronic supplementary material. The tags are able to transmit diving data and locations via satellites. Tags were programmed to transmit data every 3 days relative to the last given day.

**Table 1.** Bird groups summary (mean ± s.d.). Body condition index (juveniles only) at departure was defined as the residuals of a regression of body mass on the first axis of a principal component analysis between length of the beak and the flipper.

bird group	sample size	equipment date (±days)	monitoring duration (days)	weight at departure (kg)	body condition index
adults non-breeder	5	(1) 13 Mar 2014 (4) 23 Feb 2015 ± 3	232 ± 9 (96 ± 20)^a^	10.3 ± 1.8	—
juveniles ‘surviving’	12	(7) 14 Dec 2013 ± 14 (5) 6 Dec 2014	252 ± 34 (127 ± 28)^a^	8.8 ± 0.5	−0.02 ± 0.50
juveniles ‘early dead’	5	(4) 20 Dec 2013 ± 5 (1) 6 Dec 2014	89 ± 17 (48 ± 6)^a^	9.0 ± 1.2	0.04 ± 1.11

^a^Cumulated number of transmission days.

Three activity parameters were measured as follows: maximum dive depth, dive duration and post-dive duration. Overall, the total transmission durations of the juveniles clearly showed two distinct groups named, respectively, the ‘early death group’ (less than four months durations) and the ‘surviving group’ (more than four months durations); non-breeder adults were considered as an additional distinct group (table 1). Statistics were computed in the R3.2.1 statistical environment using linear mixed models (nlme package) with the diving parameters as response variables with ‘weeks’ and ‘years’ as fixed effects. For all models, a random slope/intercept model with weeks and individual IDs was the most parsimonious random effect structure (see electronic supplementary material, table S1). We used backward-stepwise analysis of variance for model selection (see electronic supplementary material, S1 and S2) and analysed a total of 11 840 dives for 22 individuals.

## 3. Results

**Figure 1.**
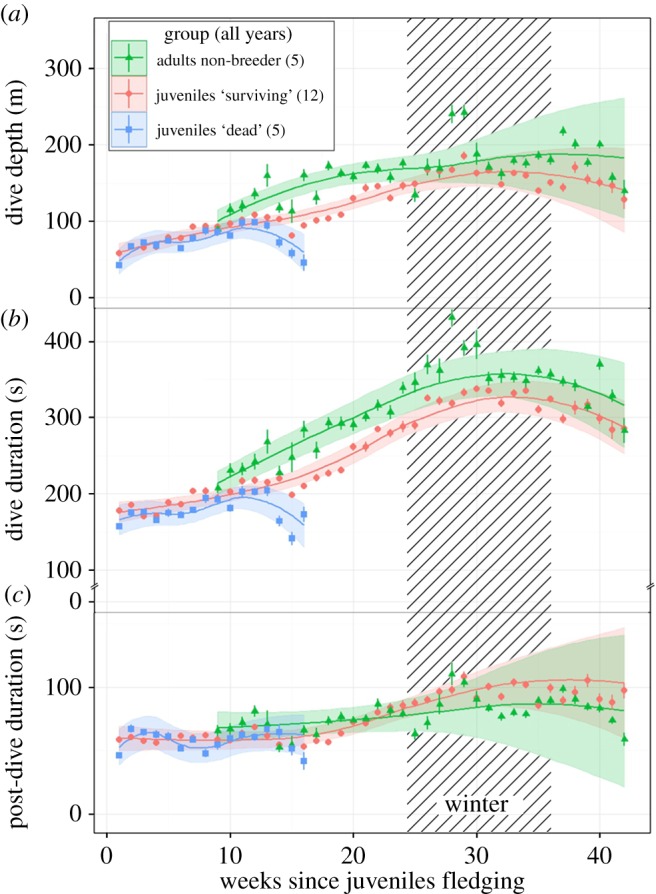
Weekly means (±s.e.) of dive depth (*a*), dive duration (*b*) and post-dive duration (*c*), with fitted value lines ±2s.e. (colour ribbon) of the best mixed models for each group (for both years). Fitted values of mixed models present a quartic effect of time for each group.

The rate of change by groups of birds differed significantly for all the three diving parameters (figure 1; electronic supplementary material, table S1). Juveniles from the ‘surviving group’ differed significantly from the adults for dive depth and duration parameters until 30 weeks but they did not differ for the post-dive duration (figure 1 and table 2). Including the factor ‘year’ in the fixed parts improved significantly the fit of the models (see electronic supplementary material, table S1) but did not change the rates of improvement of the diving parameters according to each group (see electronic supplementary material, figure S1).

**Table 2.** Estimates from the best models. Only two-way interaction parameters are shown (entire tables are available in electronic supplementary material tables S2 to S4). Dead, juveniles ‘dead’ group; nb, adults ‘non-breeder’. The reference group corresponds to the ‘surviving’ juveniles. Non-significant values are shown in italics.

parameters	dive depth			dive duration			post-dive duration		
	estimates	s.e.	*p-*value	estimates	s.e.	*p-*value	estimates	s.e.	*p-*value
week^1^:group_nb_	1183.00	459.80	0.010	1211.00	505.20	0.017	39.02	258.20	*0.880*
week^1^:group_dead_	898.30	176.00	0.000	1111.00	222.30	0.000	807.00	134.10	0.000
week^2^:group_nb_	−72.85	29.99	0.015	−65.87	32.83	0.045	−2.04	16.71	*0.903*
week^2^:group_dead_	−179.10	43.59	0.000	−213.30	52.62	0.000	−188.90	31.20	0.000
week^3^:group_nb_	2.01	0.82	0.014	1.60	0.89	*0.073*	0.11	0.45	*0.807*
week^3^:group_dead_	14.20	4.12	0.001	16.93	4.84	0.000	16.89	2.83	0.000
week^4^:group_nb_	−0.02	0.01	0.008	−0.02	0.01	*0.079*	−0.00	0.00	*0.578*
week^4^:group_dead_	−0.40	0.13	0.002	−0.50	0.15	0.001	−0.50	0.09	0.000
group_nb_:year	4.53	1.75	0.020	5.53	1.93	0.011	0.14	0.10	*0.892*
group_dead_:year	0.76	0.24	0.007	1.27	0.30	0.001	0.60	0.20	0.010

Mean depth and dive duration parameters (±s.e.) of the best models for the ‘early death group’ (figure 1) showed, after 10 weeks at sea, a decrease from 95 ± 6 m to 46 ± 11 m and from 205 ± 8 s to 172 ± 10 s. After the 35th week, the surviving group and non-breeder adults reached on average 186 ± 5 m with 337 ± 4 s, finally decreasing in spring to 129 ± 13 m and 285 ± 15 s.

Juveniles of the ‘early death group’ did not differ from other juveniles in terms of their body mass or body condition at departure (Wilcoxon tests: *W* = 31, *p* = 0.960 for both tests, table S5).

## 4. Discussion

Only a few studies have described the ontogeny of foraging behaviour in juvenile marine predators during their first year of independence [6]. We identified two groups of juveniles with variable diving capacities—one that survived and one that died. Our results showed that only the surviving group improved their diving skill while the others juveniles were not able to increase their performances. Juveniles who improved their diving skills during their first year at sea never totally reached the same performance levels as adults.

There could be several reasons behind this lower efficiency of juvenile penguins. We did not find any evidence for an association between juveniles' body condition at departure and life span or diving performance. However, at the time of their departure to sea, juveniles are smaller than adults. This smaller body size might indicate lower physiological maturity including lower myoglobin (muscle development) and haemoglobin concentration (a factor limiting O_2_ storage) and thus lower aerobic endurance [12], which could explain why juveniles have lesser diving capacities.

Adult king penguins are extremely deep divers and are able to routinely dive up to 300 m in depth and for 400 s in duration [10]. The predominant adult prey (myctophid fish [10,13]) are distributed at depths between 150 and 350 m [13]. These prey are captured mainly at the bottom of the penguins' dives. Thus, the ability of juveniles to reach deep waters is critical for accessing prey.

Differences in prey accessibility and prey quality just after fledging could also have an influence on the change in the body condition of the birds and thus on their physiological ability to dive deeper. However, the three groups used the same water masses in the Southern Ocean and there is an overlap in time and space in their foraging zones (see electronic supplementary material S3, figure S2). Moreover, adults and juveniles are thought to forage for similar prey (C.-A.B. unpublished isotopic data and prey availability in these water masses). Genetic differences in physiological capacity or incomplete maturation of cognitive capacities should also have an influence on the future capacity of the juveniles [3]. Juvenile foraging efficiency might thus be constrained by the time to learn where to find prey and how to capture it in the darkness of the deep ocean. Indeed, 5-year-old king penguins are still less efficient foragers than older birds [14]. Differences between adults and juveniles could also be due to our unbalanced sample size of adults by year. However, the diving behaviour of adult non-breeders was consistent between years (see electronic supplementary material, figure S1) and with others studies [10].

By 25 weeks at sea, the ‘surviving’ juveniles had improved their diving abilities in line with the ‘constraint hypothesis’ [2], but they had not yet reached the adults' diving capacities. This improvement enabled juveniles to exploit environmental features at a given time and depth, which is especially important as the juveniles' food intake depends on ocean resources that are unevenly distributed. Thus, a progressive increase in dive depths might suggest that juveniles continually increase their ability to attain depths where profitable prey occurs.

Later in the winter season, the continuous improvement in the diving capabilities of juveniles was associated with an increase in foraging effort, as a consequence of lower food availability [10]. Thus, juveniles were able to respond to the food scarcity and the winter migration of prey [13] by increasing their diving depth. This behaviour was also observed in adult non-breeders (figure 1), as well as in breeding king penguins [10].

In the spring, there was a subsequent decrease in the juveniles' diving parameters, confirming that juveniles adapted their behaviour to the seasonal change in prey abundance. Although the durations and depths of dives were comparable to those of the adults through the winter season, these juveniles' diving parameters were still lower, whereas they still needed the same post-dive recovery time as adults, suggesting they might be less efficient divers compared with adults, even after nine months at sea.

In contrast with surviving birds, the ‘early death group’ was characterized by poorer diving abilities. Instead of showing a progressive increase in their diving performance in the winter season, the individuals in this group showed lesser diving abilities with a progressive decrease in dive depths and durations with no change for the recovery time. These lesser diving abilities strongly suggest a mortality of individuals in this group. Birds from the ‘early death group’ seem to be unable to increase diving capacities when conditions begin to become difficult. This early mortality is likely to finally be the result of starvation and death by hypothermia, which can also be associated with predation.

To conclude, our study demonstrates that improvement in diving performances influenced survivorship during the early life of king penguins. This phenomenon may also influence not only juveniles' mortality rates in agreement with the ‘selection hypothesis’ [2], but also their dispersal and recruitment, and thus the future viability of new populations. In our rapidly changing world, juvenile king penguins could have greater difficulty in improving their diving performances and thus to survive.

## Supplementary Material

Early diving behaviour in juvenile penguins: improvement or selection processes

## Data accessibility

Raw data are deposited at: http://dx.doi.org/10.5061/dryad.6cc27 [15].
