# Supplementary material for: Early diving behaviour in juvenile penguins: improvement or selection processes
Source: Biol Lett. 2016 Aug;12(8):20160490. doi: 10.1098/rsbl.2016.0490 (PMC5014042; doi:10.1098/rsbl.2016.0490)
Supplement: Early diving behaviour in juvenile penguins: improvement or selection processes [file rsbl20160490supp1.docx]

**Early diving behaviour in juvenile penguins: improvement or selection processes**

**Florian Orgeret, Henri Weimerskirch and Charles-André Bost**

**Electronic Supplementary Material**

**S1: Statistical Protocol**

All statistics were computed with the R3.2.1 [1] statistical environment using linear mixed models (LMMs) in the nlme package (v. 3.1-122). To avoid analyzing the travelling dives thus focusing on the foraging ones, all dives less than 10 m (~37%) were removed from the analysis. Due to failed transmissions, data for some dives were missing, resulting in recorded dive durations greater than 10 min (~0.4%), which were then removed from the analysis. Furthermore, the post dive durations greater than 4 min (~3%) were excluded from the analyses in order to analyze only consecutives dives. Finally, the night dives (~17%) were also excluded from the analysis in order to focus on the foraging dives [2] and to avoid violation of the normality of the residuals due to a bimodal distribution of the depth and dives duration. Indeed juveniles and adults did not dives deeper than 70 m at night. To limit the temporal autocorrelation structure of the data, only one dive out of five were kept in the analysis. Autocorrelation of the residuals of the best models were then checked with an autocorrelation function available in R (acf()). A specific variance structure were added because of heteroscedasticity of the models’ residuals [3]. We used backward-stepwise analysis of variance for model selection with restricted maximum likelihood (REML) or maximum likelihood (ML) for random structure and fixed effects selection, respectively. For each model, one of the three diving parameters was the response variable (see equations below), the weeks were specified as a fixed linear covariate and individuals as a random effect. The overall growth curves analysis [4] of the diving parameters were modeled with fourth-order polynomials on all time term (weeks) and fixed effects of the categorical factor “groups” (“dead”, “surviving” and “non-breeders”) and year (“2014” and “2015) of individuals were added individually on all “weeks” terms. The model fits were evaluated using model comparisons based the Likelihood Ratio Test (LRT) when models were nested and on Akaike’s Information Criterion (AIC, with selection of a model when ∆AIC>2) for selection of the best variance structure. For all models a random slope/intercept model was the most parsimonious random effect structure (see table S1). The selected model was then refitted with REML prior to extraction of the model parameter estimates. We calculated an R² approximation to estimate the quality of fit of the models [5,6] using the rsquaredGLMM() function in the MuMIn package (v. 1.15.6). Graphics were plotted using the ggplot2 package (v 2.0.0) [7]. We follow the method described in [8] to calculate the body condition index of juveniles.

**S2: Equipment of Birds on the Field**

Juveniles birds were randomly selected and equipped just before their departure (November to December). Juveniles were easily recognized with their auricular patches less bright and the rest of the brow woolly plumage (especially on the head). They will moult into adult plumage at the beginning of the third year.

Non breeders birds have just finished their moult and have a new plumage some rest of few moult old feathers on the body, particularly on the head. In King penguin, the breeding patterns are more complicated than for other species of penguin because chick rearing can take up to 14 months and breeding frequency depends on breeding success in previous season; most birds probably attempt to breed annually but are only successful at maximum in 2 out of every 3 years [9].

Non Breeders were equipped from February to March and thus if they moult at this time of the year they must have succeeded their previous breeding. The precedent life history and age of this birds are not known but color of their plumage and the timing of their moult confirmed that there are adults (>5 years old). We are confident that there are as efficient as the adults breeders [10].

See [11] for the complete description of the tags and the standardized equipment method.

**S3. Models Selection.**

**Table S1. Model Selection.** **m:** model number**; df:** degrees of freedom; **LR:** Likelihood Ratio; **wk**: polynomials on week; **group**: correspond to “dead”, ”surviving” juveniles and “adults”; **pooljuvs**: dataset with “dead” and “surviving” groups’ juveniles pooled together in one group; **poolbn**: data with “surviving” group juveniles and adults pooled together in one group; **weights**: only the best variance structure is shown here: **varPower1** = (wk|ids) ; **varPower2** = (wk|group) and **varExp** = (wk|ids).

| **m** | **Model** | **random** | **weights** | **method** | **AIC** | **df** | **LR** | **pvalue** | **test** |
| --- | --- | --- | --- | --- | --- | --- | --- | --- | --- |
|  | **Dive Duration** |  |  |  |  |  |  |  |  |
| 1 | (wk+wk^2+wk^3+wk^4)*group*yr | slope \| int. |  | REML | 132353.1 | 46 |  |  |  |
| 2 | (wk+wk^2+wk^3+wk^4)*group*yr | intercept |  | REML | 132490.8 | 32 | 165.6744 | <.0001 | 1 vs 2 |
| 3 | (wk+wk^2+wk^3+wk^4)*group*yr | slope \| int. | varPower1 | ML | 131636.5 | 46 |  |  |  |
| 4 | (wk+wk^2+wk^3+wk^4)*group*yr | slope \| int. |  | ML | 132101.9 | 68 | 509.4222 | <.0001 | 3 vs 4 |
| 5 | (wk+wk^2+wk^3+wk^4)*group+group:yr+yr | slope \| int. | varPower1 | ML | 131732.6 | 56 | 120.1302 | <.0001 | 3 vs 5 |
| 6 | m3 - group:yr | slope \| int. | varPower1 | ML | 131656.6 | 66 | 24.09014 | <.0001 | 3 vs 6 |
| 7 | m3 - yr | slope \| int. | varPower1 | ML | 131647.1 | 67 | 12.63787 | 4e-04 | 3 vs 7 |
| 8 | (wk+wk^2+wk^3+wk^4)*yr | slope \| int. | varPower1 | ML | 131824.4 | 48 | 227.9188 | <.0001 | 3 vs 8 |
| 9 | (wk+wk^2+wk^3+wk^4)*group | slope \| int. | varPower1 | ML | 131748.1 | 53 | 141.5722 | <.0001 | 3 vs 9 |
| 10 | (wk+wk^2+wk^3+wk^4) | slope \| int. | varPower1 | ML | 131830.2 | 43 | 243.7117 | <.0001 | 3 vs 10 |
|  | **Dive Depth** |  |  |  |  |  |  |  |  |
| 1 | (wk+wk^2+wk^3+wk^4)*group*yr | slope \| int. |  | REML | 127903.6 | 46 |  |  |  |
| 2 | (wk+wk^2+wk^3+wk^4)*group*yr | intercept |  | REML | 128121.8 | 32 | 246.1374 | <.0001 | 1 vs 2 |
| 3 | (wk+wk^2+wk^3+wk^4)*group*yr | slope \| int. | varPower2 | ML | 127065.6 | 49 |  |  |  |
| 4 | (wk+wk^2+wk^3+wk^4)*group*yr | slope \| int. |  | ML | 127644.4 | 46 | 584.8056 | <.0001 | 3 vs 4 |
| 5 | (wk+wk^2+wk^3+wk^4)*group+group:yr+yr | slope \| int. | varPower2 | ML | 127121.2 | 37 | 79.59371 | <.0001 | 3 vs 5 |
| 6 | m3 - group:yr | slope \| int. | varPower2 | ML | 127079.0 | 47 | 17.34728 | <.0001 | 3 vs 6 |
| 7 | m3 - yr | slope \| int. | varPower2 | ML | 127069.0 | 48 | 5.361602 | 0.0206 | 3 vs 7 |
| 8 | (wk+wk^2+wk^3+wk^4)*yr | slope \| int. | varPower2 | ML | 127250.8 | 29 | 225.1167 | <.0001 | 3 vs 8 |
| 9 | (wk+wk^2+wk^3+wk^4)*group | slope \| int. | varPower2 | ML | 127119.4 | 34 | 83.79032 | <.0001 | 3 vs 9 |
| 10 | (wk+wk^2+wk^3+wk^4) | slope \| int. | varPower2 | ML | 127219.9 | 24 | 204.256 | <.0001 | 3 vs 10 |
|  | **Post Dive Duration** |  |  |  |  |  |  |  |  |
| 1 | (wk+wk^2+wk^3+wk^4)*group*yr | slope \| int. |  | REML | 117388.7 | 46 |  |  |  |
| 2 | (wk+wk^2+wk^3+wk^4)*group*yr | intercept |  | REML | 117895.5 | 32 | 534.7701 | <.0001 | 1 vs 2 |
| 3 | (wk+wk^2+wk^3+wk^4)*group*yr | slope \| int. | varExp | ML | 116944.8 | 68 |  |  |  |
| 4 | (wk+wk^2+wk^3+wk^4)*group*yr | slope \| int. |  | ML | 117249.7 | 46 | 348.9583 | <.0001 | 3 vs 4 |
| 5 | (wk+wk^2+wk^3+wk^4)*group+group:yr+yr | slope \| int. | varExp | ML | 117000.8 | 56 | 79.99013 | <.0001 | 3 vs 5 |
| 6 | m3 - group:yr | slope \| int. | varExp | ML | 116950.5 | 66 | 9.691527 | 0.0079 | 3 vs 6 |
| 7 | m3 - yr | slope \| int. | varExp | ML | 116953.2 | 67 | 10.44815 | 0.0012 | 3 vs 7 |
| 8 | (wk+wk^2+wk^3+wk^4)*yr | slope \| int. | varExp | ML | 117034.9 | 48 | 130.0879 | <.0001 | 3 vs 8 |
| 9 | (wk+wk^2+wk^3+wk^4)*group | slope \| int. | varExp | ML | 116996.8 | 53 | 81.97655 | <.0001 | 3 vs 9 |
| 10 | (wk+wk^2+wk^3+wk^4) | slope \| int. | varExp | ML | 117072.4 | 43 | 177.6108 | <.0001 | 3 vs 10 |
| 11 | (wk+wk^2+wk^3+wk^4)*poolnb*yr | slope \| int. | varExp | ML | 116970.0 | 58 | 45.22244 | <.0001 | 3 vs 11 |
| 12 | (wk+wk^2+wk^3+wk^4)*pooljuvs*yr | slope \| int. | varExp | ML | 117012.5 | 58 | 87.73417 | <.0001 | 3 vs 12 |

**Table S2. Depth Model Estimates. R^2^m** = 0.53 & **R^2^c** = 0.67; **wk1:** week^1; **wk2:** week^2; **wk3:** week^3, **wk4:** week^4; **surv:** juveniles “surviving” group; **dead:** juveniles “dead” group; **nb:** adults “non-breeder”. *The reference group correspond to the ”surviving” juveniles.*

|  | Value | Std.Error | DF | t-value | p-value |
| --- | --- | --- | --- | --- | --- |
| **(Intercept)** | 321.8 | 129.5 | 11794 | 2.486 | 0.01295 |
| **wk1** | -124.3 | 28.51 | 11794 | -4.36 | 1.31e-05 |
| **wk2** | 14.63 | 3.398 | 11794 | 4.303 | 1.695e-05 |
| **wk3** | -0.6178 | 0.1509 | 11794 | -4.095 | 4.254e-05 |
| **wk4** | 0.008628 | 0.002229 | 11794 | 3.871 | 0.0001088 |
| **groupnb** | -6631 | 2453 | 16 | -2.703 | 0.01568 |
| **groupdead** | -1046 | 326.6 | 16 | -3.202 | 0.005551 |
| **yr** | -0.2042 | 0.0956 | 16 | -2.136 | 0.04848 |
| **wk1:groupnb** | 1183 | 459.8 | 11794 | 2.573 | 0.01009 |
| **wk1:groupdead** | 898.3 | 176 | 11794 | 5.103 | 3.388e-07 |
| **wk2:groupnb** | -72.85 | 29.99 | 11794 | -2.429 | 0.01516 |
| **wk2:groupdead** | -179.1 | 43.59 | 11794 | -4.109 | 3.993e-05 |
| **wk3:groupnb** | 2.013 | 0.8169 | 11794 | 2.465 | 0.01373 |
| **wk3:groupdead** | 14.2 | 4.116 | 11794 | 3.449 | 0.0005642 |
| **wk4:groupnb** | -0.02112 | 0.008006 | 11794 | -2.638 | 0.008363 |
| **wk4:groupdead** | -0.4031 | 0.1295 | 11794 | -3.113 | 0.001859 |
| **wk1:yr** | 0.09934 | 0.0213 | 11794 | 4.665 | 3.126e-06 |
| **wk2:yr** | -0.01145 | 0.002554 | 11794 | -4.483 | 7.429e-06 |
| **wk3:yr** | 0.0004849 | 0.0001138 | 11794 | 4.261 | 2.052e-05 |
| **wk4:yr** | -6.78e-06 | 1.685e-06 | 11794 | -4.023 | 5.777e-05 |
| **groupnb:yr** | 4.532 | 1.75 | 16 | 2.59 | 0.01972 |
| **groupdead:yr** | 0.7578 | 0.2442 | 16 | 3.103 | 0.006834 |
| **wk1:groupnb:yr** | -0.8102 | 0.3283 | 11794 | -2.468 | 0.01359 |
| **wk1:groupdead:yr** | -0.6494 | 0.1315 | 11794 | -4.94 | 7.926e-07 |
| **wk2:groupnb:yr** | 0.05044 | 0.02143 | 11794 | 2.353 | 0.01862 |
| **wk2:groupdead:yr** | 0.1274 | 0.03245 | 11794 | 3.927 | 8.666e-05 |
| **wk3:groupnb:yr** | -0.001418 | 0.0005846 | 11794 | -2.426 | 0.0153 |
| **wk3:groupdead:yr** | -0.009922 | 0.003053 | 11794 | -3.25 | 0.001156 |
| **wk4:groupnb:yr** | 1.514e-05 | 5.744e-06 | 11794 | 2.636 | 0.008411 |
| **wk4:groupdead:yr** | 0.0002775 | 9.572e-05 | 11794 | 2.899 | 0.00375 |

**Table S3. Dive Duration Models Estimates. R^2^m** = 0.61 & **R^2^c** =0.65; **wk1:** week^1; **wk2:** week^2; **wk3:** week^3, **wk4:** week^4; **surv:** juveniles “surviving” group; **dead:** juveniles “dead” group; **nb:** adults “non-breeder”. *The reference group correspond to the ”surviving” juveniles.*

|  | Value | Std.Error | DF | t-value | p-value |
| --- | --- | --- | --- | --- | --- |
| **(Intercept)** | 755.1 | 155.2 | 11794 | 4.864 | 1.165e-06 |
| **wk1** | -150.6 | 37.94 | 11794 | -3.971 | 7.204e-05 |
| **wk2** | 17.46 | 4.269 | 11794 | 4.089 | 4.36e-05 |
| **wk3** | -0.6992 | 0.1849 | 11794 | -3.782 | 0.0001563 |
| **wk4** | 0.009247 | 0.002661 | 11794 | 3.474 | 0.0005138 |
| **groupnb** | -7733 | 2704 | 16 | -2.86 | 0.01134 |
| **groupdead** | -1714 | 405 | 16 | -4.232 | 0.0006351 |
| **yr** | -0.4361 | 0.1147 | 16 | -3.803 | 0.001564 |
| **wk1:groupnb** | 1211 | 505.2 | 11794 | 2.397 | 0.01653 |
| **wk1:groupdead** | 1111 | 222.3 | 11794 | 4.996 | 5.942e-07 |
| **wk2:groupnb** | -65.87 | 32.83 | 11794 | -2.007 | 0.0448 |
| **wk2:groupdead** | -213.3 | 52.62 | 11794 | -4.054 | 5.076e-05 |
| **wk3:groupnb** | 1.597 | 0.8923 | 11794 | 1.79 | 0.07348 |
| **wk3:groupdead** | 16.93 | 4.841 | 11794 | 3.498 | 0.00047 |
| **wk4:groupnb** | -0.01532 | 0.008733 | 11794 | -1.755 | 0.07933 |
| **wk4:groupdead** | -0.498 | 0.1495 | 11794 | -3.331 | 0.0008696 |
| **wk1:yr** | 0.1164 | 0.02833 | 11794 | 4.109 | 3.997e-05 |
| **wk2:yr** | -0.01342 | 0.003208 | 11794 | -4.182 | 2.913e-05 |
| **wk3:yr** | 0.0005469 | 0.0001394 | 11794 | 3.922 | 8.826e-05 |
| **wk4:yr** | -7.315e-06 | 2.013e-06 | 11794 | -3.635 | 0.0002797 |
| **groupnb:yr** | 5.534 | 1.933 | 16 | 2.863 | 0.01127 |
| **groupdead:yr** | 1.266 | 0.3028 | 16 | 4.181 | 0.0007067 |
| **wk1:groupnb:yr** | -0.8699 | 0.3617 | 11794 | -2.405 | 0.01619 |
| **wk1:groupdead:yr** | -0.8185 | 0.1658 | 11794 | -4.937 | 8.029e-07 |
| **wk2:groupnb:yr** | 0.04802 | 0.02353 | 11794 | 2.041 | 0.04126 |
| **wk2:groupdead:yr** | 0.155 | 0.03909 | 11794 | 3.966 | 7.338e-05 |
| **wk3:groupnb:yr** | -0.001187 | 0.0006406 | 11794 | -1.852 | 0.06399 |
| **wk3:groupdead:yr** | -0.01211 | 0.003581 | 11794 | -3.381 | 0.0007237 |
| **wk4:groupnb:yr** | 1.159e-05 | 6.287e-06 | 11794 | 1.843 | 0.06539 |
| **wk4:groupdead:yr** | 0.0003512 | 0.0001102 | 11794 | 3.186 | 0.001447 |

**Table S4. Post-Dive Duration Models estimates. R^2^m** = 0.19 & **R^2^c** = 0.37 ; **wk1:** week^1; **wk2:** week^2, **wk3:** week^3, **wk4:** week^4; **surv:** juveniles “surviving” group; **dead:** juveniles “dead” group; **nb:** adults “non-breeder”. *The reference group correspond to the ”surviving” juveniles.*

|  | Value | Std.Error | DF | t-value | p-value |
| --- | --- | --- | --- | --- | --- |
| **(Intercept)** | 386.5 | 107.1 | 11794 | 3.609 | 0.0003091 |
| **wk1** | -128.1 | 21.49 | 11794 | -5.962 | 2.558e-09 |
| **wk2** | 11.45 | 2.368 | 11794 | 4.834 | 1.356e-06 |
| **wk3** | -0.4133 | 0.1004 | 11794 | -4.116 | 3.876e-05 |
| **wk4** | 0.005522 | 0.001432 | 11794 | 3.856 | 0.0001157 |
| **groupnb** | -218 | 1399 | 16 | -0.1559 | 0.8781 |
| **groupdead** | -829.4 | 270.8 | 16 | -3.063 | 0.00743 |
| **yr** | -0.2451 | 0.07909 | 16 | -3.099 | 0.006896 |
| **wk1:groupnb** | 39.02 | 258.2 | 11794 | 0.1511 | 0.8799 |
| **wk1:groupdead** | 807 | 134.1 | 11794 | 6.019 | 1.813e-09 |
| **wk2:groupnb** | -2.036 | 16.71 | 11794 | -0.1218 | 0.903 |
| **wk2:groupdead** | -188.9 | 31.2 | 11794 | -6.054 | 1.456e-09 |
| **wk3:groupnb** | 0.1108 | 0.4533 | 11794 | 0.2445 | 0.8068 |
| **wk3:groupdead** | 16.89 | 2.829 | 11794 | 5.969 | 2.462e-09 |
| **wk4:groupnb** | -0.002473 | 0.004441 | 11794 | -0.5567 | 0.5777 |
| **wk4:groupdead** | -0.5037 | 0.08642 | 11794 | -5.829 | 5.72e-09 |
| **wk1:yr** | 0.09673 | 0.01605 | 11794 | 6.025 | 1.737e-09 |
| **wk2:yr** | -0.008771 | 0.001779 | 11794 | -4.931 | 8.282e-07 |
| **wk3:yr** | 0.0003224 | 7.571e-05 | 11794 | 4.258 | 2.083e-05 |
| **wk4:yr** | -4.334e-06 | 1.082e-06 | 11794 | -4.004 | 6.259e-05 |
| **groupnb:yr** | 0.1372 | 0.9979 | 16 | 0.1375 | 0.8923 |
| **groupdead:yr** | 0.5954 | 0.2023 | 16 | 2.944 | 0.00953 |
| **wk1:groupnb:yr** | -0.02583 | 0.1844 | 11794 | -0.1401 | 0.8886 |
| **wk1:groupdead:yr** | -0.58 | 0.09976 | 11794 | -5.814 | 6.252e-09 |
| **wk2:groupnb:yr** | 0.001564 | 0.01195 | 11794 | 0.1309 | 0.8959 |
| **wk2:groupdead:yr** | 0.1349 | 0.0231 | 11794 | 5.839 | 5.393e-09 |
| **wk3:groupnb:yr** | -9.239e-05 | 0.0003247 | 11794 | -0.2846 | 0.776 |
| **wk3:groupdead:yr** | -0.012 | 0.002085 | 11794 | -5.753 | 8.963e-09 |
| **wk4:groupnb:yr** | 2.018e-06 | 3.192e-06 | 11794 | 0.6324 | 0.5271 |
| **wk4:groupdead:yr** | 0.0003566 | 6.344e-05 | 11794 | 5.621 | 1.947e-08 |

**Table S5. Biometry and Body condition index.** Body condition index (juveniles only) at departure was defined as the residuals of a regression of body mass on the first axis of a principal component analysis between length of beak and flipper. **group:** **dead**: juveniles “dead” group; **surv**: juveniles “surviving” group; **nb**: adults non-breeder.

|  | **ids** | **mass (kg)** | **beak (mm)** | **flipper (mm)** | **body condition index** | **group** |
| --- | --- | --- | --- | --- | --- | --- |
| 1 | 133688 | 10.9 | 82.6 | 331 | 1.69 | dead |
| 2 | 133692 | 8.1 | 76.1 | 300 | -0.55 | dead |
| 3 | 133695 | 8.0 | 89 | 321 | -1.28 | dead |
| 4 | 133700 | 9.2 | 74.4 | 317 | 0.37 | dead |
| 5 | 141650 | 9.0 | 76 | 325 | -0.02 | dead |
| 6 | 133689 | 8.1 | 73.7 | 324 | -0.79 | surv |
| 7 | 133690 | 9.1 | 80.7 | 331 | -0.05 | surv |
| 8 | 133691 | 8.4 | 78.5 | 301 | -0.37 | surv |
| 9 | 133693 | 8.2 | 71.2 | 305 | -0.39 | surv |
| 10 | 133694 | 8.6 | 73 | 336 | -0.39 | surv |
| 11 | 133701 | 8.8 | 81 | 325 | -0.30 | surv |
| 12 | 133702 | 9.5 | 76.6 | 334 | 0.40 | surv |
| 13 | 141651 | 8.5 | 72 | 310 | -0.23 | surv |
| 14 | 141652 | 9.3 | 76 | 300 | 0.64 | surv |
| 15 | 141653 | 9.7 | 72 | 315 | 0.96 | surv |
| 16 | 141654 | 8.6 | 69 | 310 | 0.01 | surv |
| 17 | 141657 | 9.1 | 75 | 315 | 0.29 | surv |
| 18 | 133697 | 12.6 | 96.4 | 325 | x | nb |
| 19 | 141649 | 8.7 | 88.9 | 310 | x | nb |
| 20 | 141655 | 10.5 | 92.7 | 336 | x | nb |
| 21 | 141658 | 9.8 | 93.9 | 339 | x | nb |
| 22 | 141656 | 8.1 | 93.7 | 319 | x | nb |

**
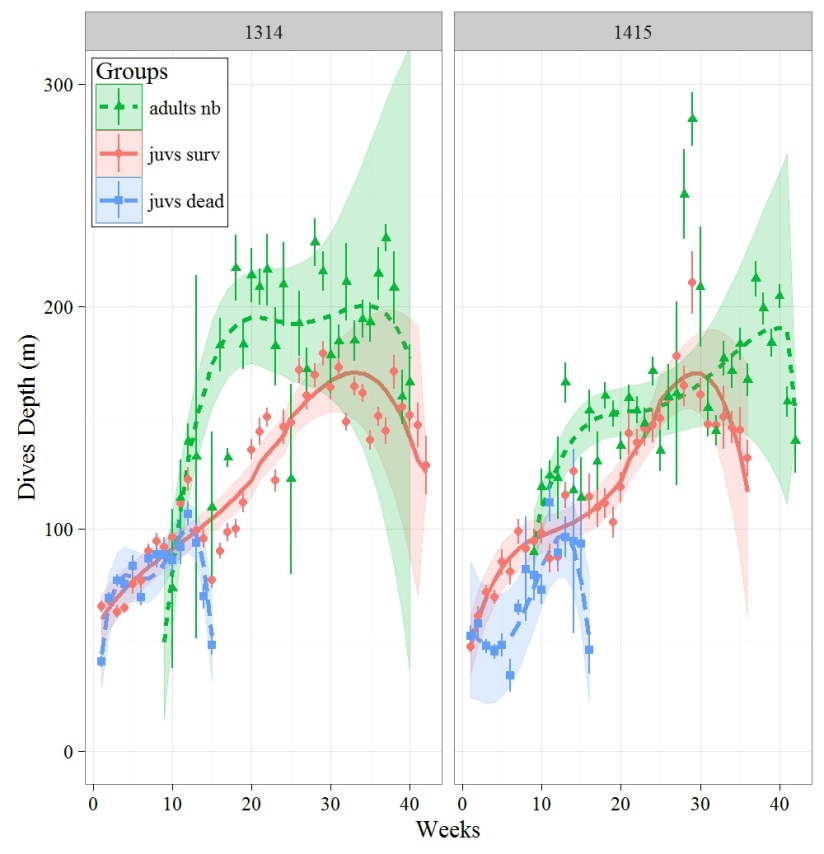
**

**Figure S1.** Weekly Mean (±SE) of Dive Depth (Right), Dive Duration (Bottom Left) and Post-Dive Duration (Bottom Right) with Fitted Values Lines ±SE for each group and for each year.

**adults nb**: adults non breeder ; **juvs surv**: juveniles surviving ; **juvs dead**: juveniles early death.

1314: year 2013/2014. 1415: year 2014/2015.


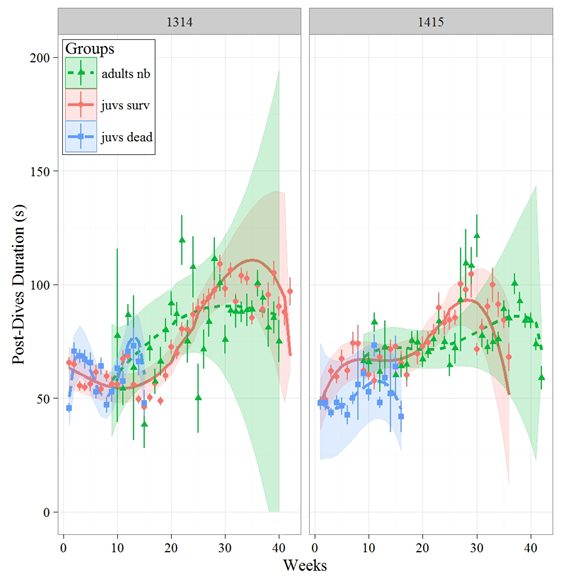
**
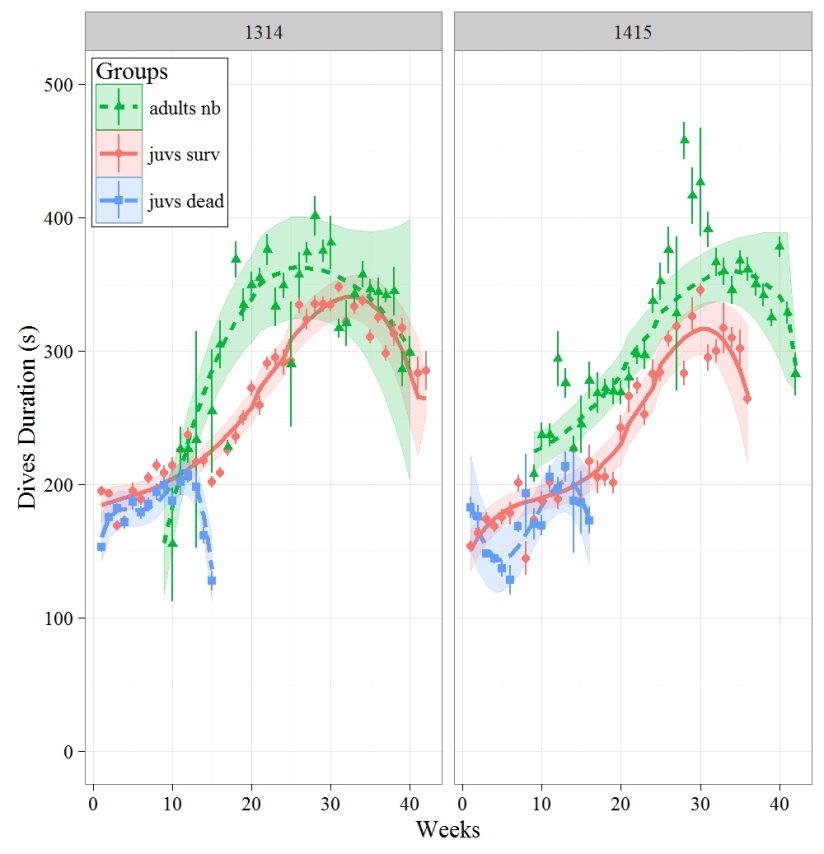
**

**S3. Kernel Maps**

Kernel analysis and map were conducted with R3.2.1 [1] statistical environment using the packages adehabitatHR (v. 0.4.14) and maps (v. 3.0.1) with the function kernelUD() which provided the Utilization Distribution of the animals and the getverticeshr() function allowing the extraction of the Home Range contours with 95% level for estimations.

The figure S2 show the Home Range of each groups in autumn, the period of time overlap between groups and also a period when food began to be scarce. Figure S2 show a great dispersion in longitude of juveniles from the “surviving” group but also a similar use of the water masses defined by the latitude and the sea surface temperature between the three groups.


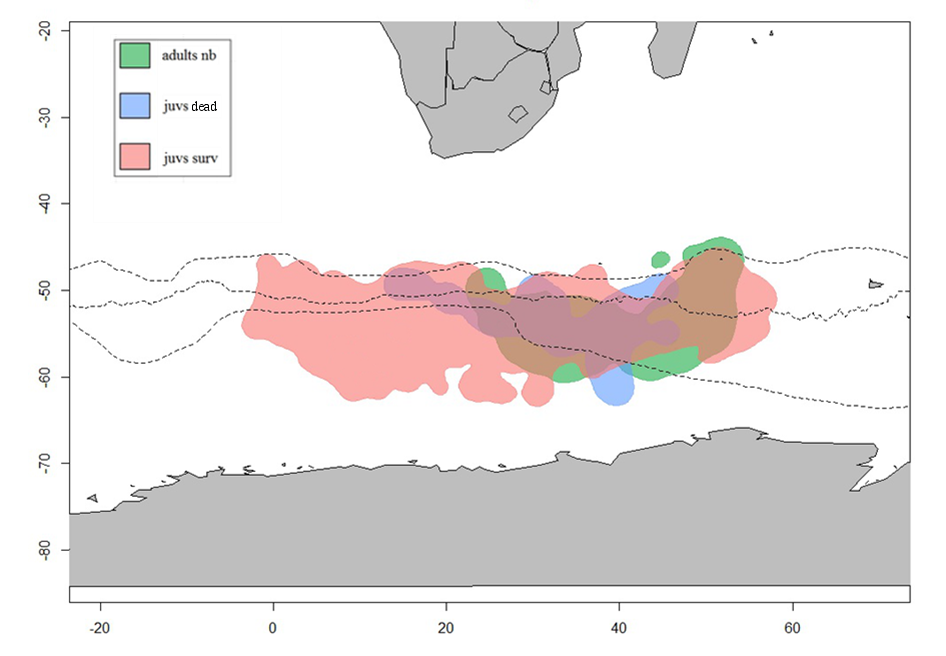


**Figure S2.** Map showing the home range contours from 95% level estimation for each groups of birds for both year, in the autumn period. The dot lines correspond to the main frontal limits in the penguins’ distribution range as indicated by the sea surface temperature [12] . **adults nb**: adults non breeder ; **juvs surv**: juveniles surviving ; **juvs dead**: juveniles early death.

**References:**

1. R Core Team 2015 R: A language and environment for statistical computing. Vienna, Austria: R Foundation for Statistical Computing (http://www.R-project.org).

2. Bost, C. A., Zorn, T., Maho, Y. L. & Duhamel, G. 2002 Feeding of diving predators and diel vertical migration of prey: King penguins diet versus trawl sampling at Kerguelen Islands. *Mar. Ecol. Prog. Ser.* **227**, 51–61. (doi:10.3354/meps227051)

3. Zuur, A. F., Ieno, E. N., Walker, N., Saveliev, A. A. & Smith, G. M. 2009 *Mixed effects models and extensions in ecology with R*. New York, NY: Springer New York.

4. Mirman, D. 2014 *Growth Curve Analysis and Visualization Using R*. CRC Press.

5. Nakagawa, S. & Schielzeth, H. 2013 A general and simple method for obtaining R2 from generalized linear mixed-effects models. *Methods Ecol. Evol.* **4**, 133–142. (doi:10.1111/j.2041-210x.2012.00261.x)

6. Johnson, P. C. D. 2014 Extension of Nakagawa & Schielzeth’s R2GLMM to random slopes models. *Methods Ecol. Evol.* **5**, 944–946. (doi:10.1111/2041-210X.12225)

7. Hadley, W. 2009 *ggplot2: Elegant graphics for data analysis*. New York: Springer.

8. Saraux, C., Viblanc, V. A., Hanuise, N., Le Maho, Y. & Le Bohec, C. 2011 Effects of Individual Pre-Fledging Traits and Environmental Conditions on Return Patterns in Juvenile King Penguins. *PLoS ONE* **6**, e20407. (doi:10.1371/journal.pone.0020407)

9. Williams, T. D. 1995 *Bird families of the world. The penguins*. Oxford University Press, New York.

10. Le Vaillant, M., Wilson, R. P., Kato, A., Saraux, C., Hanuise, N., Prud’Homme, O., Le Maho, Y., Le Bohec, C. & Ropert-Coudert, Y. 2012 King penguins adjust their diving behaviour with age. *J. Exp. Biol.* **215**, 3685–3692. (doi:10.1242/jeb.071175)

11. Thiebot, J.-B., Lescroël, A., Barbraud, C. & Bost, C.-A. 2013 Three-dimensional use of marine habitats by juvenile emperor penguins *Aptenodytes forsteri* during post-natal dispersal. *Antarct. Sci.* **25**, 536–544. (doi:10.1017/S0954102012001198)

12. Bost, C. A., Cotté, C., Bailleul, F., Cherel, Y., Charrassin, J. B., Guinet, C., Ainley, D. G. & Weimerskirch, H. 2009 The importance of oceanographic fronts to marine birds and mammals of the southern oceans. *J. Mar. Syst.* **78**, 363–376. (doi:10.1016/j.jmarsys.2008.11.022)
